# The Role of Hedgehog Signaling in Tumor Induced Bone Disease

**DOI:** 10.3390/cancers7030856

**Published:** 2015-08-26

**Authors:** Shellese A. Cannonier, Julie A. Sterling

**Affiliations:** 1 Department of Veterans Affairs, Tennessee Valley Healthcare System, Nashville, TN 37235, USA; E-Mail: shellese.a.cannonier@vanderbilt.edu; 2 Vanderbilt Center for Bone Biology, Department of Medicine, Division of Clinical Pharmacology Vanderbilt University, Nashville, TN 372335, USA; 3 Department of Cancer Biology, Vanderbilt University, Nashville, TN 37235, USA

**Keywords:** Hedgehog signaling, Gli, cancer, bone, metastasis

## Abstract

Despite significant progress in cancer treatments, tumor induced bone disease continues to cause significant morbidities. While tumors show distinct mutations and clinical characteristics, they behave similarly once they establish in bone. Tumors can metastasize to bone from distant sites (breast, prostate, lung), directly invade into bone (head and neck) or originate from the bone (melanoma, chondrosarcoma) where they cause pain, fractures, hypercalcemia, and ultimately, poor prognoses and outcomes. Tumors in bone secrete factors (interleukins and parathyroid hormone-related protein) that induce *RANKL* expression from osteoblasts, causing an increase in osteoclast mediated bone resorption. While the mechanisms involved varies slightly between tumor types, many tumors display an increase in Hedgehog signaling components that lead to increased tumor growth, therapy failure, and metastasis. The work of multiple laboratories has detailed Hh signaling in several tumor types and revealed that tumor establishment in bone can be controlled by both canonical and non-canonical Hh signaling in a cell type specific manner. This review will explore the role of Hh signaling in the modulation of tumor induced bone disease, and will shed insight into possible therapeutic interventions for blocking Hh signaling in these tumors.

## 1. Introduction

Hedgehog (Hh) signaling is a complex signaling pathway that was initially identified as a developmental signaling pathway. Since its original discovery in 1980, its role in disease has been better defined. Aberrant Hh signaling has been demonstrated by numerous groups to contribute to multiple cancer types both through contributing to changes in tumor growth directly or altering the tumor microenvironment. Because of its role in multiple tumor types, Hh signaling is an attractive pathway for both basic research and drug development to identify new targets and new therapeutic approaches for inhibiting tumor growth and metastasis.

## 2. Hedgehog Signaling in Skeletal Development and Bone Homeostasis

### 2.1. Hedgehog Signal Transduction

Hh signaling, first identified by its developmental role in *Drosophila melanogaster*, is an evolutionary conserved signaling pathway that is now known to have additional important roles in tissue homeostasis as well as tumorigenesis [1,2,3,4]. In the late 1960s, the field of embryonic development had no dogma to explain how the relatively simple body plan of *drosophila* embryos gave rise to the complex body structure of adult fruit flies. In the 1970s, Christiane Nüsslein-Volhard and Eric Wieschaus made ground breaking progress in understanding drosophila tissue patterning and polarity, which they were later awarded for with the Nobel Bell Prize in physiology or medicine. Utilizing embryonic lethal screening techniques, the pair introduced random mutations into the genome of fruit flies using ethyl methanosulfonate (EMS) and identified 15 loci important for segment number and polarity in drosophila larvae [5]. The resulting developmental defects were mainly named after the phenotypic observations of the larvae. In the case of Hh, the phenotype affected the denticles, which are bristle hairs used for gripping surfaces and locomotion. While normal embryos displayed discrete bands of denticles, Hh mutants showed an unorganized “denticle lawn” reminiscent of the spines of the hedgehog, hence the name Hh. Further research in the field identified Hh signaling as an important morphogen, where levels of secreted ligand creates a short span gradient that directly controls genes important for cell proliferation, polarity, and differentiation [6,7,8,9,10]. 

Additionally, in context specific settings, Hh signaling has been found to function in tandem with other important developmental signaling pathways such as Wnt, BMP and TGFβ signaling [10,11,12]. Crosstalk between these pathways facilitates multiple levels of transcriptional control over target genes and as such, Hh signaling can both positively and negatively regulate other signaling pathways. Hh signaling has an active and inactive state, which is controlled by the presence of Hh ligand. In insects, when there is no Hh ligand bound to the receptor Patched (Ptch), the membrane protein Smoothened (Smo) is unable to accumulate and its effector molecule, the zinc finger protein, cubitus interruptus is proteasomally cleaved into a transcriptional repressor (CiR). In contrast, Hh ligand bound to Ptch leads to Smo accumulation which prevents Ci cleavage, allowing the full length protein to function as a transcriptional activator [13].

**Figure 1:**
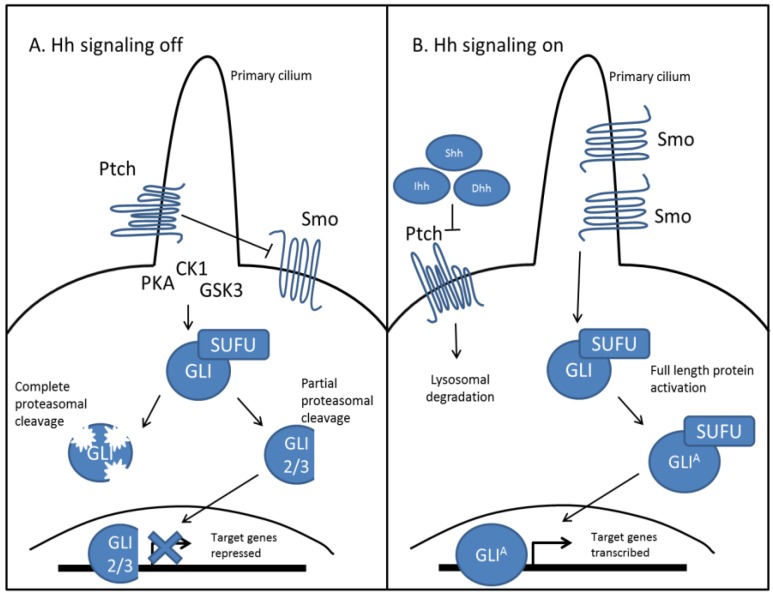
Canonical Hedgehog signaling in mammals is ligand dependent. (**A**) In the absence of Hh ligands, Ptch accumulates in the primary cilium and inhibits the function of Smo. Ptch facilitates the activation of several kinases (CK1, PKA, GSK3), at the base of the primary cilium, which differentially phosphorylate Gli protein. This can lead to complete degradation of Gli protein by the proteasome, as well as partial cleavage of Gli2 and Gli3. Partially cleaved Gli is translocated to the nucleus and functions as a transcriptional repressor for Hh target genes; (**B**) In the presence of Hh ligands, Shh, Ihh, or Dhh binds to Ptch, which induces its lysosomal degradation. This relieves its inhibitory effect on Smo, which accumulates in the primary cilium and prevents degradation of Gli proteins. Activated Gli protein is translocated to the nucleus and functions as a transcriptional activator for Hh target genes.

As an evolutionarily conserved signaling pathway, Hh signaling works similarly in vertebrates, as outlined in Figure 1 [14]. Of the three mammalian Hh ligands, Sonic Hh (Shh), Indian Hh (Ihh), and Desert Hh (Dhh), the most studied and well understood ligand remains Shh. Binding of the Hh ligand to Ptch relieves its inhibitory effect on Smo, which then accumulates in the primary cilium and through a complex signaling cascade, facilitates the recruitment and activation of Gli proteins, which are the mammalian homologues of Ci [15]. Once active, these proteins are translocated to the nucleus, where they function as transcription factors and upregulate their target genes. Importantly, activation of Hh signaling leads to expression of both Ptch and Gli1, where Ptch expression creates a negative feedback loop to regulate both levels and duration of Hh signaling [16]. Gli1 expression is used to amplify target genes, as it functions exclusively as a transcriptional activator [17]. Conversely, in the absence of Hh ligand, Gli proteins are complexed with several binding proteins in the cytosol. Ptch mediated activation of several kinases (CK1, PKA, GSK3) lead to Gli phosphorylation, which in the case of Gli1, targets it for complete proteasomal degradation. Gli3 and to some extent, Gli2 are processed into repressors via proteasome-mediated carboxyl cleavage [18,19]. These repressors are translocated to the nucleus where they downregulate Hh target genes, which include genes important for proliferation (Cyclin D1/D20), cell survival (BCL2), and epithelial to mesenchymal transition (EMT) (Snail1) [20,21]. Hh target genes include a myriad of regulators which include both oncogenes and tumor suppressors, giving rise to a diverse and interconnected signaling system, that when dysregulated can lead to a host of diseases including cancer [22,23,24,25]. 

### 2.2. Hedgehog Signaling in Skeletal Development 

In vertebrates, both growth and ossification of the skeletal system is achieved via Hh signaling. Early in development, cellular patterning of the limb bud is controlled predominantly by Shh. Acting as a classical morphogen; secretion of Shh forms a spatial and temporal gradient which controls cellular differentiation, polarization, and proliferation [26]. In contrast, Ihh is the predominant ligand governing Hh signaling for its role in bone formation. Proper endochondral ossification is essential for long bone formation. Ihh controls endochondral ossification through a dynamic feedback loop that also controls growth plate development [27]. In this process, Ihh secretion from prehypertrophic chondrocytes at the ends of the bones induces expression of parathyroid hormone-related protein (*PTHrP*) in periarticular chondrocytes [28,29,30]. *PTHrP* is secreted and diffuses along the growth plate, which increases chondrocyte proliferation in the growth plate region, and induces them to deposits large amounts of collagenous extra-cellular matrix (ECM), extending the length of the developing bone. Beyond this region, levels of *PTHrP* drop and chondrocytes no longer proliferate but instead undergo hypertrophy and then apoptosis [31]. Following, the resulting matrix and empty space is invaded by vasculature which is followed by osteoblast mediated mineralization, completing ossification. 

Loss of Ihh during bone development has been shown to have detrimental effects on bone elongation and ossification [27]. One group demonstrated that loss of Ihh leads to premature chondrocyte hypertrophy, resulting in significantly shortened limbs lacking ossification. Additionally, levels of osteoblasts were also significantly reduced [32,33]. Several groups have demonstrated that Hh signaling is critical for osteoblast differentiation, which is important for chondrogenesis and cartilage vascularization and thus endochondral ossification [34,35,36]. Intramembranous ossification also requires Hh signaling. Ihh null mice present with underdeveloped calvaria that show reduced ossification [27,37]. Work done to understand this phenotype has demonstrated that Ihh regulates intramembranous ossification via osteogenic differentiation, and to some extent, proliferation [38]. Additionally, in these models, Ihh levels regulate BMP2/4 expression, suggesting that these pathways cooperate to control intramembranous ossification [38]. These findings appear to be central to Hh signaling, as partial loss of Gli3, which mainly functions as a repressor for Hh signaling, leads to increased ossification of calvarial bone. In these mouse models it was observed that loss of one Gli3 allele led to craniosynostosis of the lambdoid sutures in the skull [39,40]. Collectively, this information supports the essential role of Hh signaling in bone development.

### 2.3. Hedgehog Signaling in Bone Homeostasis

Post-pubescent skeletons undergo constant, albeit low levels of remodeling. The two main cell types that maintain bone homeostasis are osteoblasts and osteoclasts. Osteoblasts are differentiated bone forming cells derived from mesenchymal stem cells, while osteoclasts are differentiated bone resorbing cells derived from monocytes. These cell populations have directly opposing functions that regulate one another in a tightly controlled feedback loop [41]. Osteoblasts express receptor activator of NFkB ligand (*RANKL*) on their cell surface which binds to *RANK* on pre-osteoclasts and facilitates their differentiation. As these cells differentiate, they fuse into large multinucleated cells. Differentiated osteoclasts secrete metalloprotein enzymes that are tartate resistant, and are known as tartate resistant acid phosphatase (TRAP) positive cells [42]. Other hallmarks of a functional osteoclast include a “zone of attachment” or sealing zone, where the plasma membrane of the cell adheres to the bone surface through use of integrin mediated podosomes and a resorption pit, the space directly under the osteoclast. The ruffled border, which increases the surface area of the cell that can contact the bone, as well as Cathepsin K secretion are also required for resorption. Cathepsin K is a protease known to catabolize collagen as well as elastin. Combined with the acidic gradient produced in the resorption pit, TRAP, Cathepsin K, and other secreted enzymes, such as matrix metalloproteinases (MMPs) degrade the bone ECM, allowing dissolution of the inorganic component of bone, hydroxyapatite [43]. Importantly, bone resorption releases and activates large amounts of growth factors and cytokines, which are normally sequestered in the ECM. These released signaling molecules have several effects, including contributing to osteoblastogenesis and inducing osteoprotegerin (OPG) expression. OPG is a soluble receptor for *RANKL*, known as a decoy receptor because it binds free *RANKL* and therefore blocks *RANK*/*RANKL* binding, thus preventing further osteoclastogenesis. This coupling of osteoblast activity to osteoclast activity allows for balanced levels of bone remodeling, where bone is resorbed at about the same rate that it is made [44]. 

**Figure 2:**
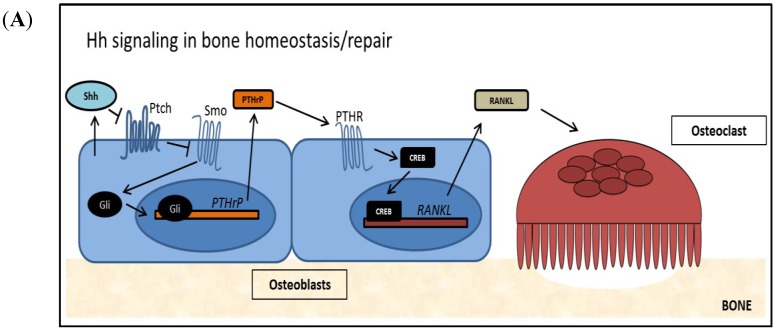

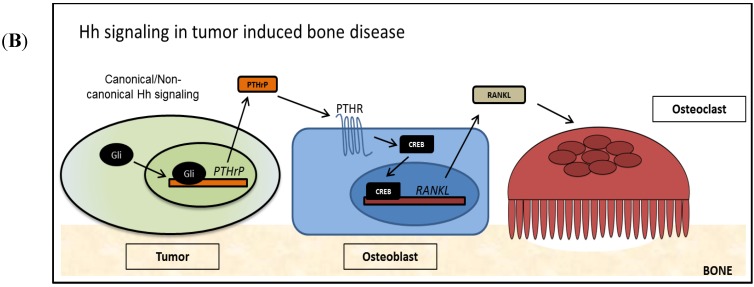
The Role of Hh Signaling in Bone (**A**) Hh signaling is active in normal bone remodeling and repair. Osteoblast derived Hh ligand activates Hh signaling cell autonomously as well as non-cell autonomously. *PTHrP*, a target of Hh signaling, is transcribed and *PTHrP* is secreted from the cell. Secreted *PTHrP* binds to its receptor PTHR on the surface of osteoblasts and activates the transcription factor CREB. *RANKL*, a target of *PTHrP*/*PTHR* signaling is expressed and *RANKL* is presented at the cell surface of osteoblasts, where it induces osteoclastogenesis and supports bone resorption; (**B**) Tumor-derived Hh signaling disrupts normal bone remodeling and induces TIBD. Tumor cells re-activate Hh signaling by canonical and/or non-canonical mechanisms, increasing Gli activity and Hh target genes. *PTHrP is* over expressed in these cells, and a large amount of *PTHrP* is secreted, which activates the *PTHR* receptor on osteoblasts. Following, large amounts of *RANKL* are produced by the osteoblasts, which lead to increased osteoclastogenesis and excessive bone resorption.

Defects in Hh signaling have been shown to play an important role in bone remodeling. Evidence for this includes the finding that Ptch1 deficient patients show increased bone mass due to increased osteoblast differentiation [45]. Conversely, in a mouse model utilizing activated Hh signaling in mature osteoblasts, bone density and quality were shown to be significantly reduced. This was found to be caused indirectly; in mature osteoblasts Hh signaling induces *PTHrP* expression which upregulates *RANKL*, increasing osteoclastogenesis [46]. This finding is physiologically relevant, as Shh is found to be upregulated in mature osteoblasts at fracture sites. Hh signaling in mature osteoblasts has been shown to be key for several processes, such as osteoblast proliferation and differentiation, as well as healing by mediating vascularization [47,48,49]. As outlined in Figure 2A, Hh signaling in mature osteoblasts leads to expression and secretion of *PTHrP*, which induces expression and secretion of *RANKL*. *RANKL* is required for osteoclastogenesis. Mature osteoclasts cause an increase in osteoclast mediated resorption of old and/or damaged bone, which is required to facilitate healing and normal bone turnover. 

## 3. Hedgehog Signaling in Tumorigenesis and Tumor Induced Bone Disease (TIBD)

### 3.1. Hedgehog Signaling in Tumorigenesis 

Hedgehog signaling has been implicated in several stages of tumorigenesis [50]. The first major finding supporting this came from studying Gorlin syndrome [51,52,53,54]. Patients with Gorlin syndrome present with several skeletal abnormalities, including intracranial calcification, abnormal rib and spine curvature, and cranio-facial defects [55]. This condition is also known as nevoid basal cell carcinoma syndrome as patients often develop basal cell carcinoma (BCC). Research in genetics has identified the cause of this syndrome. A microdeletion of chromosome 9q causes a loss-of-function mutation in the *PTCH1* gene [56,57]. This leads to inadequate levels and/or a mutated non-functional version of Ptch, both of which causes haploinsufficiency. In mouse models heterozygous for *PTCH1*, the murine skeletal abnormalities mirror those of humans [58]. In addition, UV damage is sufficient to induce BCC in mice heterozygous for *PTCH1* [59]. It is thought that the ultra violet radiation causes mutations that render the remaining *PTCH1* allele non-functional. This supports the finding that sequenced BCC tumors often show *PTCH1* deletions, as well as the clinical observations of BCC being most commonly diagnosed on the face, arms, back and chest; areas commonly exposed to sunlight [60,61]. Patients heterozygous for *PTCH1* have an increased risk for other tumors as well [62]. Keratocystic odontogenic tumors of the jaw are most common but are benign and are rarely life-threatening. Other tumors, including rhabdomyosarcoma and fibromas can occur. While Gorlin syndrome is an inherited autosomal dominant disorder that causes loss of the tumor suppressor gene *PTCH1*, it only facilitates tumor development if the function of the other allele is disrupted. However, sporadic BCC and medulloblastoma (MB) frequently occur in patients with both *PTCH1* alleles because discrete cellular alterations cause Hh signaling to become constitutively active. This often occurs genetically through mutations that directly inactivate Ptch or activate Smo, but can also occur indirectly if mutations affecting downstream regulatory proteins cause an upregulation of Hh target genes [53,63,64,65,66]. Several studies in MB have supported this finding by identifying mutations in *SUFU*, *GLI1*, and *GLI2* in tumors, but not in normal surrounding stroma [67,68].

There are several other tumors types that show dysregulation in Hh signaling. For example, *GLI1* is named glioma-associated oncogene homolog1 for its association with glioblastoma multiforme (GBM) [69]. Although aberrant Hh signaling has not been found to be a driver mutation in forming these tumors, there remains a role for Hh signaling in other stages of GBM tumorigenesis [70,71,72]. Several groups have shown activated Hh signaling in gliomas, which sustains survival and stemness of glioma cells [73,74,75]. Additionally, Hh inhibition using cyclopamine has been shown to reduce glioma tumorigenicity [73,75,76]. Together these data support the role of Hh signaling in maintaining the tumorigenic potential of glioblastoma.

In breast and prostate cancer, activated Hh signaling is correlated with recurrence, metastasis, and ultimately lower overall survival [77,78,79,80,81,82]. In breast cancer, Hh signaling components play several roles. Over-expression of Hh ligands is associated with a basal-like phenotype that leads to poor prognosis in patients [77]. Similarly, activated Gli1 protein correlates with higher risk of recurrence after surgery [77,78,79]. Importantly, Hh signaling can be activated as a result of signaling cooperativity with a number of other signaling pathways [83,84,85,86,87,88,89,90,91]. For example, in breast cancer cells responsive to estrogen (ER+), estrogen stimulation induces *GLI1* expression, which is important for invasion and cell renewal [92]. In breast cancer cells that acquire resistance to anti-estrogen therapies, Hh signaling remains activated downstream of the receptor via the PI3K/AKT pathway [93]. Additionally, Hh ligand secreted from the tumors can stimulate Hh signaling in the tumor cells, but the microenvironment also plays an important part in responding to secreted Hh ligand [94]. The stimulated stromal cells in the microenvironment cause dynamic changes that can facilitate tumor progression by inducing vascularization and migration associated genes [95]. Thus in breast cancer, activated Hh signaling can support tumorigenesis by facilitating tumor progression as well as modulating the microenvironment to a pro-tumorigenic state. 

Hedgehog pathway activation is also observed in prostate cancer, where activation can be detected in both the tumor and the stroma [96,97,98]. Hh signaling has been found to become upregulated in prostate cancer cells when they are deprived of androgen for long periods of time [99]. Additionally, Hh signaling remains activated in androgen-independent cells [100,101]. Research done by Chen and colleagues demonstrate that upregulated Hh signaling supports androgen signaling by enhancing androgen specific gene expression [101,102]. Other tumor types that use cooperative signaling of Hh signaling and other pathways to promote tumorigenesis include pancreatic cancer, malignant melanoma, gastric/colon cancer, lung and ovarian cancer, which are well outlined in this recent review [103]. 

### 3.2. Hedgehog Signaling in Tumor Induced Bone Disease 

Advanced stage tumors, as well as aggressive tumors have an increased propensity to metastasize to distant sites of the body. Along with the lung and the liver, the bone is a common site of metastasis, where tumors in the bone disrupt normal bone remodeling and cause TIBD [104]. In patients, TIBD causes bone pain, hypercalcemia, and an increased risk of fracture, along with reduced skeletal mass and bone lesions [105]. Many tumor types, including breast, prostate, and lung, metastasize to bone via the circulatory system, while other tumors such as head and neck cancer invade directly into the facial bones from surrounding tissues. Primary bone tumors, such as osteosarcoma and multiple myeloma, also cause bone disease. While the molecular mechanisms controlling bone disease varies by tumor type, all of the above mentioned tumors dysregulate normal bone remodeling and induce excessive bone resorption in a process coined “The Vicious Cycle” [106]. In the vicious cycle, tumor cells that arrive at the bone microenvironment respond to chemical and physical cues by secreting factors that stimulate bone resorption. Cytokines such TGFβ and BMP present due to normal bone remodeling can stimulate tumor cells [107]. Additionally, our group has shown that the rigidity of the bone matrix can stimulate gene expression via mechanotransduction signaling [108]. As outlined in Figure 2B, stimulated tumor cells, in a cell type specific manner, re-activate Hh signaling, which can be achieved by activating mutations in canonical Hh signaling receptor proteins Smo and Ptch, or non-canonically through other signaling pathways that lead to Gli activation such as TGFβ, PI3K/AKT, ERK and others. Increased Gli levels lead to expression and secretion of parathyroid hormone-related protein (*PTHrP*), which increases *RANKL* production in osteoblasts, stimulating osteoclastogenesis. Additional signaling factors such as interleukins 6/8/11, and TNF-α, also increase osteoclastogenesis in a similar manner, but have not been shown to be directly dependent on Hh signaling [109,110,111,112]. Ultimately, as an increased number of osteoclasts mature and resorb bone, the released growth factors from the ECM further stimulate tumor cells, which causes a positive feedback loop. Tumor cells in the bone microenvironment uncouple osteoblast/osteoclast signaling, and lead to increased bone resorption as well as impair bone quality. 

In breast cancer metastases to bone, this process is mediated by Hh signaling. Using bone metastatic MDA-MB-231 breast cancer cells, we have shown that TGFβ stimulation increases expression of Gli2 and *PTHrP*, which controls bone destruction [112]. In an experimental model of metastasis, tumor cells injected into the left cardiac ventricle home to the skeleton where they proliferate and induce bone destruction. We show that when Gli2 expression is repressed using a plasmid over-expressing Gli2 repressor, both *PTHrP* expression and bone destruction are significantly reduced [113]. In this system, Gli activity does not seem to be regulated canonically, as bone tropic MDA-MB-231 cells do not express the Smo receptor nor do they respond to cyclopamine treatment [112]. A similar observation can be seen in malignant melanoma. Interestingly, while the melanoma cell lines used showed Smo and Ptch gene expression, Gli2 was found to be a transcriptional target of TGFβ signaling. Using 1205Lu melanoma cells that express high levels of Gli2, the authors showed that loss of Gli2 using shRNA led to a decreased incidence of bone metastases as well as smaller lesions [114].

Not surprisingly, primary bone tumors such as chondrosarcoma and osteosarcoma also dysregulate bone remodeling. Chondrosarcoma tumors originate from cartilage and are thought to arise due to activating mutations in Hh signaling [115]. Studies have shown that many primary chondrosarcomas express all canonical Hh signaling proteins, as well as *PTHrP* [115,116,117,118,119]. Using these tissues in primary organ cultures, one group showed that Hh signaling was constitutively active due to loss of *PTCH1*/2, activation of Smo or a combination of both. Additionally, using the Hh inhibitor, triparanol, in a xenograph mouse model, the authors observed decreased proliferation, cellularity, and tumor size [115]. 

Aberrant Hh signaling inducing TIBD is also observed in head and neck tumors. Oral squamous cell carcinoma (OSCC), which accounts for almost 90% of all head and neck tumors show activated Hh signaling as measured by immunohistochemical staining of Hh proteins in patient biopsies [120]. Levels of Hh proteins also correlate with poor prognosis [121]. A subset of OSCC patients are affected by mandibular invasion, where the tumor directly invades the mandible through underlying tissue and begins to proliferate in the bone, stimulating bone destruction [122,123]. One group in Japan demonstrated that loss of Shh in OSCC cells decreased tumor volume as well as bone destruction in an *in vivo* model of bone destruction [124]. Here tumor cells stably expressing shRNA against Shh were inoculated into the tibia of immunocompromised mice. Our group has also observed OSCC dependency on Hh signaling. We utilized an orthotopic model of mandibular invasion and bone destruction, where tumor cells are injected into the masseter muscle and invade directly through underlying tissues into the mandible. Using this model, we injected human OSCC cells stably expressing shRNA against Gli2 and observed that loss of Gli2 decreased both bony invasion and bone destruction in male athymic mice. These studies highlight the importance of Hh signaling on TIBD and suggest that Hh signaling components are viable targets to prevent TIBD.

## 4. Potential Therapeutics to Target Hh Signaling Components

Due to its role in tumorigenesis, Hh signaling inhibitors are a promising potential therapy to inhibit tumor growth and disease, and, thus, have been tested as anti-tumor agents extensively [125,126,127]. The natural plant alkaloid cyclopamine was the first compound identified as an inhibitor of Hh signaling [128]. In animal models, cyclopamine demonstrated great potential and was found to inhibit tumor growth in several tumor types, including BCC, MB, breast, and pancreatic cancer [81,90,129,130,131,132,133]. Unfortunately, use of cyclopamine *in vivo* revealed that the drug had low oral bioavailability, a short elimination half-life, and serious adverse effects due to toxicity [134]. However, with the development of synthetic and semi-synthetic compounds, as well as the identification of additional inhibitors using high throughput screening, Smo has been shown to be an excellent druggable target. Vismodegid, TAK-441, IPI-926, Saridegib, Sonidegib/Erismodegib, BMS-833923/XL139, PF-04449913, Taladegib/LY2940680, and CUR61414 represent a newer class of small molecule inhibitors with increased potency for Smo and an improved pharmacokinetic profile [135,136,137,138,139,140,141,142,143,144,145,146,147,148]. Several clinical trials have demonstrated the efficacy of these compounds, especially in tumor types with activating mutations in Hh signaling due to Smo [142,143,144,147,149,150,151,152]. In January 2012, the FDA approved Vismodegib for use in locally advanced and metastatic BCC, after a Phase II clinical trial showed response rates over 30% [149]. Even so, patients on Smo inhibitors relapse due to acquired mutations in Smo or reactivation of Hh signaling downstream through other signaling pathways affecting Gli activity [88,91,141,153,154,155,156,157,158]. Unfortunately, these patients relapse in a matter of months and fail to respond to additional Smo inhibition. While therapies to target drug-resistant tumors are being developed, inhibition of Hh signaling by targeting downstream modulators is actively being investigated [159]. Importantly, tumors that show resistance to Smo inhibitors retain sensitivity to Gli inhibitors and when treated, significant reductions in Hh target genes are observed [160].

**Figure 3:**
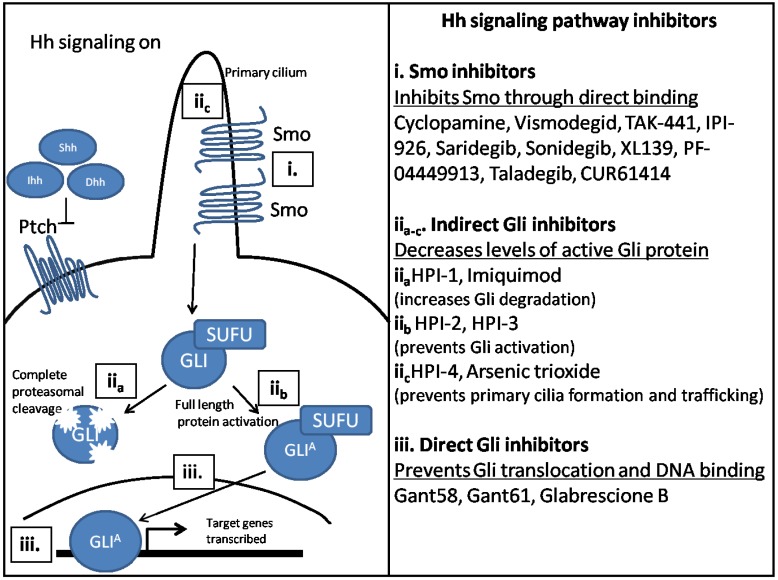
Inhibitors of the Hh signaling pathway target different Hh signaling components. (i) Smo is the first identified target for Hh inhibition and remains the best studied. Smo inhibitors function by binding directly to Smo and preventing downstream activation; (ii) Indirect Gli inhibitors inhibit Hh signaling downstream of Smo, but have differing mechanisms. HPI-1 and Imiquimod increase Gli degradation (ii_a_), while HPI-2 and HPI-3 prevent Gli protein activation (ii_b_). HPI-4 inhibits ciliogenesis, while arsenic trioxide prevents Gli trafficking to the primary cilium (ii_c_); (iii) Direct Gli inhibitors inhibit Hh signaling by binding directly to Gli protein and inhibiting its transcriptional activity. GANT58 prevents Gli translocation to the nucleus. GANT61 and Glabrescione B inhibit transcription of Hh target genes, by interfering with the DNA binding pocket of Gli protein.

Thus, the field has now transitioned to developing Hh inhibitors that target Hh signaling downstream of Smo. Gli inhibition has been proposed as the ultimate target, as it is the effector molecule for Hh signaling. Inhibitors of Hh signaling that indirectly affect Gli are also being investigated. Gli inhibition would enable downregulation of Hh signaling in non-canonical mechanisms of Hh signaling activation, such as PI3K activation. Inhibitors that directly and indirectly inhibit Gli have been identified, and are described in Figure 3. Gli inhibitors are the most attractive as drug targets because they are most downstream on the Hh signaling pathway, decreasing the likelihood of Hh reactivation. 

The Gli antagonists, GANT58 and GANT61, were the first small molecule inhibitors determined to inhibit Hh signaling at the level of Gli protein [161]. Recently, another Gli inhibitor, Glabrescione B has been identified [162]. Glabrescione B binds directly to the DNA binding site of Gli1 while GANT61 binds indirectly, but still prevents Gli mediated transcription [162,163]. GANT58 in contrast, prevents Gli translocation to the nucleus [161]. GANT61 has been shown to inhibit tumor growth in several mouse models of tumor burden [163,164,165]. Additionally, Glabrescione B has been shown to be efficacious in decreasing tumor burden in tumors over-expressing Hh signaling components [162]. While these drugs show great promise both *in vitro* and in pre-clinical studies, it remains unclear if any are potential candidates for clinical trials. 

Four Hh pathway inhibitors (HPI-1-4) have been identified by Hyman and colleagues [166]. These compounds inhibit processing and trafficking of Gli protein and each inhibitor has been found to have a distinct mechanism of action. HPI-1 causes an increase in repressor forms of Gli, while HPI-2 and HPI-3 both prevent Gli protein activation [166]. HPI-4 inhibits ciliogenesis, preventing both Gli accumulation and processing in the primary cilium [166]. Imiquimod, which is used to treat BCC, also inhibits Hh signaling downstream of Smo [4,167]. Known as an agonist of Toll-like receptor 7/8, imiquimod activates PKA, which induces increased degradation of Gli proteins. Arsenic trioxide is another inhibitor of Hh signaling, which is used for treatment of acute promyelocytic leukemia [168]. This drug prevents Gli trafficking to the primary cilium, thus preventing its activation. Newly emerging studies, investigating the use of epigenetic silencing and indirect inhibitors to target Hh signaling have been conducted [169,170,171,172,173,174,175]. 

Currently, there have been several murine studies exploring the efficacy of a variety of Gli inhibitors in different tumor models [176,177,178,179]. While the use of Gli inhibitors have been promising, the greatest challenge that remains is the lack of understanding molecular mechanisms surrounding Gli mediated transcription, especially in the context of other active oncogenic pathways. This setback has made Hh inhibition in TIBD especially challenging. Tumors that metastasize to and establish in bone represent a subset of the primary tumor with differential expression of tumorigenic signaling pathways. In the case of breast, prostate, and lung cancer, Hh signaling is known to contribute to tumor progression and a stem-cell like phenotype, but is not found to be a driving mutation. While work from our group demonstrates the importance of Gli on bone destruction in breast to bone metastasis, additional research is required to understand mechanisms in other tumor types. However, based on the increasing number of studies involving Gli inhibition to target Hh signaling, the field is primed to move forward in identifying novel Gli inhibitors as well as elucidating mechanisms controlling dysregulated Hh signaling in cancer progression. 

## 5. Conclusions

Due to its role in tumor progression, metastasis, and treatment failure in many different tumor types, Hh signaling remains an appealing target for the development of therapeutics. Despite its promise in pre-clinical and early clinical studies, Smo inhibition alone is no longer considered an effective treatment for the inhibition of cancer growth, due to patient relapse and resistance. Because of this, combination therapies are currently being tested to evaluate efficacy in several different tumor types [180,181,182,183,184,185,186,187]. Additionally, several pre-clinical compounds targeting downstream of Smo and specifically Gli show great potential, but have proven challenging. Important remaining questions in the field are two-pronged. The first seeks to identify Gli inhibitors that are superior to Smo inhibitors, but are feasible as therapeutics in Hh driven cancers. The second is focused on identifying pragmatic applications of Gli inhibitors in preclinical models of tumorigenesis to prevent progression. This second point is especially important, as information concerning drug mechanisms, pharmacokinetic profiles as well as short-term/long-term side effects is still largely unknown in many tumor types. As additional novel inhibitors are discovered and designed, a better understanding of how Hh mechanisms change in response to inhibition will be required to help refine existing therapies to show more clinical success.
